# Dynamic Assembly of Human Salivary Stem/Progenitor Microstructures Requires Coordinated α_1_β_1_ Integrin-Mediated Motility

**DOI:** 10.3389/fcell.2019.00224

**Published:** 2019-10-16

**Authors:** Danielle Wu, Robert L. Witt, Daniel A. Harrington, Mary C. Farach-Carson

**Affiliations:** ^1^Department of Diagnostic and Biomedical Sciences, The University of Texas Health Science Center at Houston, Houston, TX, United States; ^2^Department of BioSciences, Rice University, Houston, TX, United States; ^3^Center for Translational Cancer Research, Helen F. Graham Cancer Center & Research Institute, Christiana Care Health Center, Newark, DE, United States; ^4^Department of Otolaryngology-Head and Neck Surgery, Thomas Jefferson University, Philadelphia, PA, United States

**Keywords:** basement membrane, integrin, mechanotransduction, salivary gland, human, stem/progenitor cells

## Abstract

A tissue engineering approach can provide replacement salivary gland structures to patients with hyposalivation disorders and xerostomia. Salivary human stem/progenitor cells (hS/PCs) were isolated from healthy regions of parotid glands of head and neck surgery patients, expanded, then encapsulated in biocompatible hyaluronate (HA)-based hydrogels. These bioactive hydrogels provide a surrogate territorial matrix suitable for the dynamic assembly, growth and reorganization of salivary gland components. This study examined the dynamics of salivary microstructure formation, growth, and reorganization using time-lapse imaging over 15 h. Immunofluorescence detection monitored production of individual basement membrane components forming around developing microstructures, and Ki67 assessed proliferation. Dynamic movements in hydrogels were quantified by measuring angular velocity (ω) of rotating salivary microstructures and changes in basement membrane architecture during microstructure growth. Integrin involvement in the dynamic reassembly was assessed using knockdown and inhibitor approaches. Single hS/PCs expanded over 5 days into spherical microstructures typically containing 3–10 cells. In larger macrostructures, proliferation occurred near the peripheral basement membrane that underwent growth-associated cycles of thinning and collapse. *De novo* secretion of laminin/collagen IV from reorganizing hS/PCs preceded that of perlecan/HSPG2. Microstructures routinely expressed β_1_ integrin-containing complexes at basement membrane-associated regions and exhibited spontaneous and coordinated rotation during basement membrane maturation. β_1_ integrin siRNA knockdown at the single-cell state prevented hS/PC microstructure growth. After microstructure formation, β_1_ integrin knockdown reduced rotation and mean ω by 84%. Blockade of the α_1_ integrin subunit (CD49a) that associates with β_1_ reduced mean ω by 66%. Studies presented here show that initial hS/PC structure growth and basement membrane maturation depends on α_1_β_1_-integrin mediated signaling. Coordinated cellular motility during neotissue reorganization reminiscent of salivary gland acini was critically dependent both on hS/PC-secretion of laminin,collagen type-IV, and perlecan/HSPG2 and the force-driven interactions of α_1_β_1_-integrin activation. We conclude that α_1_β_1_-integrin plays a critical role in establishing human salivary gland coordinated structure and function, and that its activation in tissue engineered systems is essential to tissue assembly.

## Introduction

The long-term focus of work in our lab is to devise tissue engineering approaches to fully reestablish salivary function in patients suffering from hyposalivation disorders related to cancer treatment, aging, disease, or injury. Salivary gland engineering is a challenge because the gland is a complex physiological system in which multiple cell types, including several distinct epithelia, comprise an intact functional gland. These epithelia natively form in direct contact with the underlying mesenchyme, which provides biological cues to direct differentiation and development. This work builds upon observations in salivary gland development that point to the critical role of the α_1_β_1_-integrin and associated extracellular matrix (ECM) in salivary morphogenesis (Larsen et al., 2006; Daley et al., 2011). We investigated the dynamic reorganization of primary human salivary stem/progenitor cells (hS/PCs) that we characterized previously (Srinivasan et al., 2017) after encapsulation within a biomimetic hyaluronate (HA) hydrogel designed to mimic native tissue mesenchyme. The hydrogel culture system offers optical clarity, preservation of 3D architecture, and control of the microenvironment representing the natural mesenchyme that surrounds a developing exocrine gland, allowing us to explore the early events that occur during hS/PC reorganization as they mimic events in salivary gland morphogenesis.

In tissue, the basement membrane is the link between the epithelium and the underlying mesenchyme, providing orientation and organization, allowing selective filtration of nutrients and waste, and establishing survival cues. Integrins, present on epithelial cells including those in salivary glands (Togarrati et al., 2018), initiate cytoskeletal reorganization and associated cell signaling processes in development, homeostasis, and regeneration. Adhesion-dependent processes enhance responses to growth factors present in the environment to initiate cellular processes (Chen et al., 1994; Miyamoto et al., 1996) including cellular proliferation and differentiation, signal transduction activation, cell survival signals, and gene expression occurring as early as 6 h post-adhesion (Royce et al., 1993; Hoffman et al., 1996; Bouhadir and Mooney, 1998; Lafrenie et al., 1998; Overeem et al., 2015). Integrin ligands in the basement membrane including laminin (Virtanen et al., 2000), collagen IV, and perlecan/HSPG2 surround ductal and acinar compartments in salivary glands. Each component of the basement membrane has unique functions. Laminin directly interacts with integrins (Nishiuchi et al., 2006) expressed by epithelial cells to induce cell polarity and organization of developing structures and tissues (Li et al., 2003; Patel et al., 2006; Daley et al., 2012). Collagen IV fibers in the basement membrane interact primarily with the underlying mesenchyme in connective tissue to provide tensile strength critical for regulating integrin turnover during dynamic cell movements and tissue maintenance (Pines et al., 2012). Another critical component, perlecan/HSPG2, is essential because of its complex repertoire of adhesive motifs (Yurchenco et al., 1987; Hayashi et al., 1992; Battaglia et al., 1993; Brown et al., 1997; Ettner et al., 1998), its ability to serve as a growth factor depot (Chen et al., 2007), and its innate compressive strength and flexibility as a proteoglycan with long flexible core and hydrophilic glycosaminoglycan chains (Wijeratne et al., 2016). Perlecan also mechanically stabilizes the basement membrane (Farach-Carson et al., 2014), because perlecan's core protein anchors to laminin while its heparan sulfate chain containing domains integrate with collagen IV in a “spot-weld” fashion (Behrens et al., 2012).

We hypothesized that dynamic interactions between integrins present on hS/PCs and their matrix ligands in the basement membrane mediate salivary hS/PC microstructure growth and dynamic reorganization. Using a variety of techniques including quantitative time lapse imaging, immunostaining, RNA knockdown, and direct inhibition, we investigated the role of α_1_β_1_-integrin in early events during hS/PC microstructure formation and basement membrane assembly. The temporal and spatial organization of *de novo* microstructures was analyzed to reveal how physical and biological morphogenic cues direct salivary gland architecture, reorganization, and growth dynamics, all needed to support development of tissue engineered replacements to provide a permanent solution for hyposalivation disorders.

## Materials and Methods

### Human Subjects Research

This study was carried out in accordance with the recommendations of the Christiana Care Health System Institutional Review Board (IRB)-approved protocols with written informed consent from all subjects. All subjects gave written informed consent in accordance with the Declaration of Helsinki. The protocol was approved by the IRBs at CCHS, Rice University and UTHealth as well as by the Committee for the Protection of Human Subjects at UTHealth.

### Tissue Culture

Patients undergoing scheduled surgery at Christiana Care Health System (Newark, DE) consented for an unaffected portion of their parotid gland tissue to be transferred to Rice University or the University of Texas Health Science Center at Houston under IRB-approved protocols. The fresh parotid gland tissue was prepared in agreement with a standard operating protocol for generating hS/PCs (Wu et al., 2018). hS/PCs were cultured in Hepato-STIM™ medium supplemented with 10 ng/mL EGF (355056; Corning) and 1% (v/v) penicillin-streptomycin (15140122; Life Technologies/ThermoFisher), and maintained at 37°C in a 5% (v/v) CO_2_ incubator as described previously (Pradhan et al., 2009). The studies in this article used samples from three female donors age, 22, 57 and 63. hS/PCs expressed biomarkers, K5, K14, and p63 and were encapsulated in hydrogels and cultured in complete Hepato-STIM™ medium for these studies. A full characterization of these cells appeared in Srinivasan et al. (2017) and they were fully sequenced in the functional annotation of the mammalian genome 5 (FANTOM5) project (FANTOM Consortium the RIKEN PMI CLST et al., 2014).

### Encapsulation and Hydrogel Culture

Early passages (between 3 and 6) of hS/PC cells were encapsulated at 3 × 10^6^ cell/mL in HyStem® hydrogel (GS311; BioTime/Ascendance Biotechnology). According to manufacturer's instructions, hydrogels were formed by mixing reconstituted thiol-modified hyaluronic acid (5.9 mM) and polyethylene glycol diacrylate (1.5 mM) at a 4:1 volume ratio, and plated on microscope glass slides fitted with pre-sterilized arrays of 50 μL wells made from laser-cut polydimethylsiloxane (PDMS; Sylgard™ 184; Dow Corning) sheets (Figure S1). Hydrogels were removed from the mold then each transferred into individual wells of a 48-well plate and cultured as described above. A typical hydrogel formed a low-profile cylinder measuring 6 mm in diameter and 2 mm in height. Hydrogels also were formed directly on confocal glass bottom dishes (P50G1.54F; MatTek) for live-cell imaging. 3D cultures were maintained until microstructures reached up to ~50 μm in diameter (between 6 and 16 days), a time when most live-tracking experiments were initiated. Viability assays using Calcein AM (80011-3; Biotium) and Ethidium Homodimer-III (40050; Biotium) in accordance with manufacturer guidelines were performed in cell-laden hydrogel cultures. IMARIS 9.2 software (Bitplane) was used to separate and quantify objects (Movie S1).

### Immunocytochemistry

Human parotid gland tissue cryosections embedded in CRYO-OCT Compound (Tissue-Tek) or cells encapsulated in hydrogels were fixed with 4% (w/v) paraformaldehyde (A11313; Alfa Aesar), permeabilized with 0.2–3% (v/v) Triton® X-100 (A16046; Alfa Aesar), and blocked with 3–10% (v/v) goat serum (5058835; EMD Millipore). Samples were incubated with primary antibodies against basement membrane proteins: laminin (NB300-144; Novus Biologicals, 1:200), collagen IV (NBP1-97716, 1:200; Novus Biologicals), perlecan/HSPG2 (NBP1-05170, 1:200; Novus Biologicals), or β_1_ integrin (NB100-63255, 1:200; Novus Biologicals), followed by appropriate secondary antibodies conjugated to Alexa Fluor 488 (A11029; Life Technologies/ThermoFisher), Alexa Fluor 568 (A11011; Life Technologies/ThermoFisher) to tag proteins of interest. Nuclear [4′,6-diamidino-2-phenylindole (DAPI); 40011; Biotium] and filamentous actin (A22287; Life Technologies/ThermoFisher) counterstains were used prior to mounting (P36930; Life Technologies/ThermoFisher), if necessary, and confocal imaging (A1; Nikon Instruments) of specimens.

### Western Blots

Cells transfected with siRNA in 6 well-plate were harvested with and lysed in RIPA buffer containing: 150 mM NaCl, 5 mM EDTA, 1% (v/v) NP-40, 24 mM sodium deoxycholate, 3.5 mM SDS, and supplemented with protease inhibitors (Halt™ Protease Inhibitor Cocktail (100X), 78430; Thermo Scientific). Proteins were separated by SDS-PAGE and transferred to nitrocellulose membranes for immunoblotting with antibodies. Primary antibodies were monoclonal mouse anti-integrin β1 (NB100-63255, 1:500, Novus Biologicals) and polyclonal rabbit GAPDH (PA1-980, 1:1000; Invitrogen). Secondary antibodies were horseradish peroxidase-conjugated sheep anti-mouse (515-035-062, 1:25,000) or goat anti-rabbit (111-035-144, 1:50,000) from Jackson ImmunoResearch Laboratories Inc. For β_1_ integrin knockdown studies in 2D, cells were either transfected with scrambled siRNA or ITGB1 siRNA and collected at 72 h post-transfection.

### Time-Lapse Imaging and Image Analysis

For brightfield live imaging experiments, hydrogels were transferred to glass bottom culture dishes and placed within a temperature controlled heated stage set to 37°C during 15 h live-imaging experiments (Eclipse TE300 and A1R MP+ microscopes, Nikon Instruments). Angular velocities (ω) (rev/h) of organizing hS/PC microstructures (diameters of 20–52 μm) were calculated manually from time-lapse imaging videos where images were acquired every 5 min. Time-lapse recordings and confocal micrographs were analyzed using NIS Elements AR (Nikon Instruments). Dimensional and positional information was extracted from individual frames in each time-lapse recording. For fluorescence image analysis, fluorescence intensities were divided into four groups: I = 0, I > 500, I > 1000, and I > 3000. The baseline-background intensities (I_b_) were calculated from confocal images of secondary only control hydrogels used in the analysis and are 188 ± 4 (A.U.), 221 ± 2 (A.U.), and 48 ± 2 (A.U.) for laminin, collagen IV, and perlecan, respectively. Timestamps of fluorescence micrographs or movies of hS/PCs in hydrogels will be in the form of days (D) or hours. Dimensional, colocalization, and surface analysis of fluorescence images was performed on confocal micrograph stacks using IMARIS 9.2 (Bitplane). Image preparation and visualization required NIS Elements AR, IMARIS, Fiji (Schindelin et al., 2012), ImageJ (NIH), and Adobe Photoshop (Adobe).

### Integrin Analysis

Published literature was consulted to identify the integrin subunits reported to be present in normal human salivary tissue. The summary of these findings is presented in Table S1, along with key studies establishing binding interactions between integrins and their ligands expressed in human salivary tissue: laminin, collagen IV, and perlecan/HSPG2. This analysis implicated the β_1_ integrin as a likely player in salivary reassembly, thus immediate efforts were focused on this integrin subunit. Integrin subunits that form dimers with β_1_ integrin expressed in human salivary tissue include α_1_, α_3_, α_5_, and α_6_. Integrin subunits α_3_ and α_6_ are expressed at lower levels than α_1_, and α_5_ is essential during branching morphogenesis via fibronectin interactions. The integrin subunit α_1_ is abundant in the epithelial compartments of the salivary gland and a prime candidate for interacting with basement membrane components secreted by hS/PCs: laminin, collagen IV, and perlecan/HSPG2, during early reorganization.

### Small Interfering RNA and Transfections

Knockdown of the expression of β_1_ integrin was accomplished using an siRNA approach (*Silencer*® Select siRNA, 4390825; Life Technologies/ThermoFisher). ITGB1 siRNA (s7574) labeled with Cy3 (AM1632; Ambion), or *Silencer*® Cy^TM^3-labeled Negative Control No. 1 siRNA (AM4621; Ambion) was used to identify structures transfected with siRNA or scrambled siRNA. Cells in hydrogels were transfected with a final concentration of 192 nM of each siRNA sequence under rotating culture conditions using Lipofectamine RNAiMAX Transfection Reagent (13778150; Invitrogen) for 6 h. Quantification of knockdown using siRNA targeting ITGB1 in hS/PC microstructures (hydrogels) and in monolayers (6-well plate) was, respectively, performed by immunocytochemistry 46 h post-transfection and Western blot 72 h post-transfection. A coverage index (*I*_*C*_) in Equation 1 was developed to quantify the amount of β_1_ integrin expression surrounding a microstructure to evaluate the effectiveness of siRNA treatment in hydrogels.

(1) $$\begin{array}{l} I_{C}=\;\frac{A_{\beta 1\;integrin}}{A_{s}} \end{array}$$

*I*_*C*_ is dimensionless and defined by the area occupied by β_1_ integrin at the surface of the microstructure (*A*_β1__*integrin*_) normalized to the theoretical surface area (*A*_*s*_) in Equation 2 of the microstructure determined by respective microstructure radii.

(2) $$\begin{array}{l} A_{s}=4\pi r^{2} \end{array}$$

*A*_β1__*integrin*_ is quantified from confocal z-stacks using the IMARIS image analysis software. Surfaces are generated from immunofluorescence intensities of β_1_ integrin expression for microstructures treated with scrambled siRNA (*n* = 10) or ITGB1 siRNA (*n* = 10). Calculations of *A*_β1__*integrin*_ are generated and normalized by *A*_*s*_ to determine *I*_*C*_ for both treatment groups (*n* = 10). *A*_β1__*integrin*_ universally was greater since surface area calculations for each β_1_ integrin plaque were summed and contributed to an observed inflation of *I*_*C*_. However, relative differences remain true since each confocal z-stack was subjected to the same image analysis algorithm.

### Loss of Function Studies

Loss of function studies were conducted between 3 and 7 days after encapsulation, to ensure structure sizes were similar at the time of treatment and time-lapse imaging studies were performed within 36 h post-transfection. Treatments were performed the same way for all hydrogels regardless of encapsulated cell/cluster size. The ω of Cy3-labeled ITGB1 siRNA^+^ microstructures (*n* = 37) compared to Cy3-labeled scrambled siRNA controls (*n* = 25) was measured.

Next, CD49a blocking antibody (anti-CD49a, Clone 5E8D9, NBP2-29757; Novus Biologicals) was used to stabilize the inactive conformation of β_1_ integrin subunits with their α_1_ subunit partners, effectively blocking functional interactions with matrix ligands (Forsyth et al., 2002). Culture media without blocking antibody as well as a non-blocking CD49a antibody (anti-CD49a, Clone CL7207, NBP2-76478; Novus Biologicals) served as controls. CD49a antibodies (25 μg/mL) were added for 1 h prior to time-lapse imaging to allow full diffusional access to cells in hydrogels (Du et al., 2011). The ω of antibody-blocked structures moving in hydrogels (*n* = 32) was calculated and analyzed as described above and compared to no treatment (*n* = 24) and non-blocking (*n* = 14) controls.

### Statistical Analysis

All quantified values are shown as mean ± SEM, with *n* = number of events. Unpaired Student's *t*-tests were used to identify differences between control and treated groups. One-way ANOVA followed by a post-test was used to identify differences between groups >2. No significant differences were identified when comparing cells from three donors (*p* = 0.55) (Figure S2), so data from control and treated conditions from all sources was analyzed together to increase sample size. Asterisks indicate significant differences when compared with reference controls (^***^p < 0.001). Prism 7.0 (Graph Pad) was used for statistical analyses.

## Results

### Cell Encapsulation and Microstructure Formation

**Figure 1:**
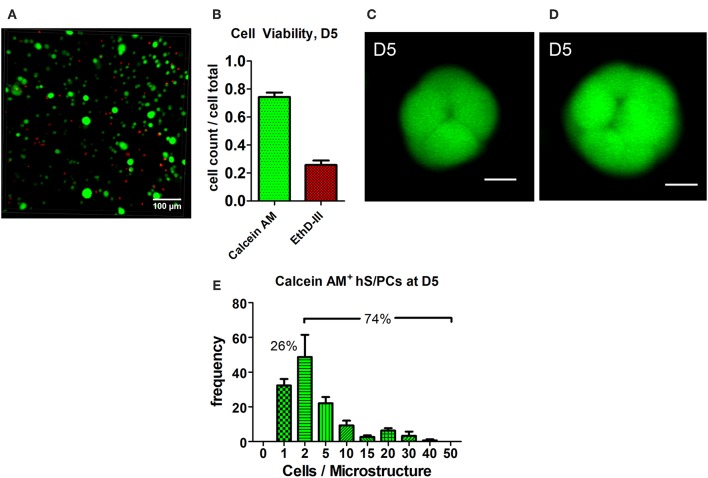
Cluster formation and analysis of microstructure size. hS/PCs cultured for 5 days in HA-based hydrogels formed clusters up to 35 μm in diameter **(A)** with 74.3% viability **(B)** determined by Live/Dead assays. Confocal micrographs were used to quantify cell viability from regions of interest (ROI) of equivalent volumes. Organizing microstructures of 30–35 μm containing 8 and 16 cells, respectively (**C,D**, scale bar = 10 μm), are readily seen. The distribution of the number of hS/PCs within each microstructure encapsulated in HA-based hydrogel at D5 was measured with respect to microstructure diameter. Approximately 74% of viable cells were in clusters and 26% of viable cells remained single cells **(E)**.

Encapsulated hS/PCs in HA-hydrogel systems readily formed multicellular 3D microstructures. Most cell death observed at day 2 (D2) occurred during the encapsulation process or in the period immediately after encapsulation. EthD-III^+^ dead cells remained as single cells and had not divided nor co-localized with other viable single-cells or clusters (not shown). These non-viable cells at D2 post-encapsulation accounted for 24% of cells, and they remained in the single-cell state. By day 5 post-encapsulation (D5), 74.3 ± 0.3% of the total viable cells were either clustered or remained at the single-cell state (Figures 1A,B). Furthermore, all single hS/PCs after 5 days in culture express laminin and 32% express perlecan (Figure S3), when a representative region of interest is evaluated. Two examples of viable microstructures at D5 had diameters of 30 and 37 μm and contained 8 and 16 cells, respectively (Figures 1C,D). Analysis of the total viable population showed that cluster volumes at D5 averaged 9465 ± 985 μm^3^ with mean diameters of 26 μm and containing 5–6 cells. Of the viable cells, ~74% were in clusters and 26% remained as single cells (Figure 1E). In Figure 1E, values within each group are binned to include quantities between an interval beginning and including the group value to and excluding the larger group value, i.e., group 1 contains the cell count between 1 <2, including 1 but excluding 2. Structure sizes varied throughout the entire hydrogel, and did not track with location. Larger structures (>40 μm in diameter) were found distributed throughout the hydrogel as well as in the hydrogel center, indicating that nutrient diffusion was not limited near the center of the hydrogel.

### Dynamics of Basement Membrane Formation

**Figure 2:**
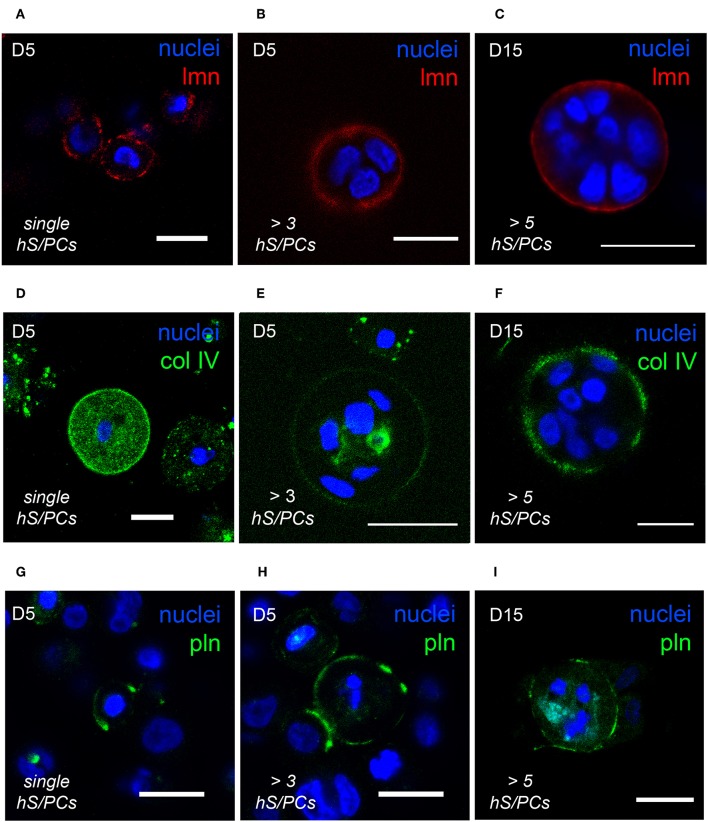
hS/PCs sequentially secrete basement membrane proteins during microstructure reorganization and function. Encapsulated hS/PCs in HA-PEGDA hydrogels initially produce laminin (red) **(A–C)** and collagen IV (green) **(D–F)** even at the single-cell state **(A,D)**. Perlecan (green) **(G–I)** secretion follows to stabilize the laminin and collagen IV networks. Scalebar = 20 μm in all confocal micrographs.

The optical clarity of HA-based hydrogels made visualization of dynamics of basement membrane formation in 3D feasible. Single-cell and multicellular motility of cells and microstructures was captured using time-lapse microscopy and production of basement membrane components in time-matched specimens was analyzed using immunocytochemistry (ICC). We initially observed that the interactions involved in hS/PC microstructure formation, growth and basement membrane maturation preceded polarization and lumen formation that occurred only later in larger structures. The synthesis and secretion of basement membrane proteins began shortly after encapsulation in cultured hS/PCs in 3D HA-hydrogel cultures, evident even in single cells (Figures 2A,D,G). Encapsulated hS/PCs at the single-cell state initially secreted basement membrane proteins laminin (Figure 2A) and collagen IV (Figure 2D), and some perlecan/HSPG2 (Figure 2G) at the periphery to form *de novo* basement membranes surrounding larger individual salivary microstructures. All single hS/PCs in culture express laminin and 32% express perlecan when evaluated after 5 days in culture (Figure S3), and shows laminin is routinely deposited at the single-cell state. Collagen IV was seen in both the secretory pathway and at the periphery in single cells and larger clusters (Figures 2D–F). Both laminin and collagen IV surrounded larger structures (Figures 2B,C,E,F), but perlecan expression was discontinuous at the structure periphery, regardless of cluster size (Figures 2H,I). Laminin, collagen IV, and perlecan proteins surrounding single hS/PCs and hS/PC microstructures were quantified with respect to structure diameters and fluorescence intensities (Figure S4). Basement membrane proteins are present at early and later growth stages. Laminin and collagen IV are secreted by single hS/PCs by D5 (Figures S5A–C) and by D15, laminin remains at the periphery of the multicellular cluster accompanied by perlecan (Figures S5D–F). Production of individual basement membrane components was similar to that seen in native tissue, where laminin, collagen IV, and perlecan surround and separate individual acini, co-located with β1 integrin on the basal cell surfaces of acinar cells (Figure S6).

### Microstructures “Spin” as They Deposit Basement Membrane

**Figure 3:**
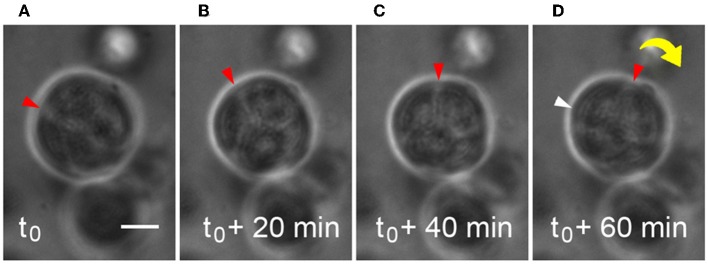
Microstructure dynamics during rotation in HA-based hydrogel. Movement of a 30 μm microstructure was measured and found to rotate ~92°/h during basement membrane deposition. Still frames are extracted from time-lapse movies of microstructure motility at 20 min intervals **(A–D)**. Scale bar = 10 μm.

Microstructures containing two or more cells were observed to generate enough traction to begin to rotate or “spin” in the HA-based hydrogels during the same time period when deposition of basement membrane components was occurring (Movie S2). Adhesive interactions between integrins on cells and matrix or hydrogel networks surrounding them can generate traction forces large enough to drive observed revolutions of entire multicellular structures, and this behavior was observed in Figures 3A–D. Cells within microstructures moved together in a coordinated manner yielding a net directional rotation whose angular velocity (ω) could be measured. Microstructures were observed to move both in a clockwise or a counterclockwise fashion, even within the same gel. The coordinated motion of cells within microstructures was quantified by their rotation where ω was calculated from time-lapse imaging micrographs. In cases where the surrounding *de novo* basement membrane was not completely formed and/or exhibited border irregularity, neither the rotation nor coordination were quantifiable. Therefore, only angular velocities of smooth, multicellular structures exhibiting coordinated rotations were included in the analysis. Analysis of 118 spinning structures meeting these criteria showed that untreated microstructures 33.9 ± 1.06 μm in diameter rotated at 0.28 ± 0.02 rev/h, *n* = 49. An example of this is shown in Figure 3 where a 30 μm microstructure rotates at 92°/h (ω = 0.26 rev/h).

If cells in a microstructure were seen to move independently from one other within its structure, net rotations were never observed. This lack of coordination was seen periodically in clusters of 3–4 cells during early stages of organization. Immunocytochemistry revealed cell-secreted laminin and collagen IV surrounded individual cells within microstructures of comparable size (not shown).

### Growth of Microstructures After Basement Membrane Formation

**Figure 4:**
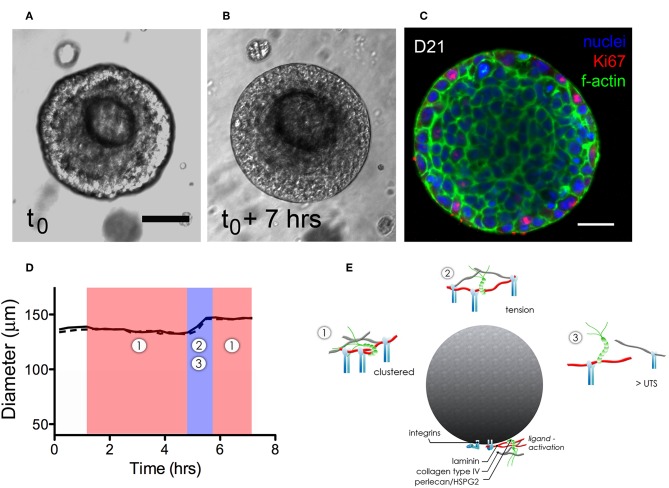
Dynamics of expansion of an hS/PC microstructure in HA-based hydrogel. Non-linear structural expansion was observed over a 7 h period **(A,B)**. Total expansion increased structure volume by 33%. Ki67 staining revealed proliferative cells at the periphery of the microstructure, adjacent to the basement membrane **(C)**. Dimensional analysis measuring major and minor axes (x-dotted line, y-solid line) in **(D)** reveals microstructure contraction during phase 1 followed by a growth and tensile phase (2–3) for <1.5 h that visibly stretched and thinned the basement membrane. Schematics of phases 1–3 in **(E)** focus on integrin-basement membrane interactions. During phase 1, integrins are more tightly spaced when associated with laminin and collagen IV in the basement membrane. Expansion-induced tension on the basement membrane in phase 2 increased periodicity of in-tact integrin attachments reaching tensions large enough to break perlecan spot-welds between laminin and collagen IV networks. Ultimate tensile strength (UTS) of proteins in the basement membrane are approached during phase 3-expansion causing reorganization and subsequently remodeling of the basement membrane as contractile events during phase 1 began. Scalebar = 25 μm.

Dynamic growth and organization of microstructures that had formed in hydrogels were tracked using time-lapse imaging to identify how various dynamic processes affected microstructure growth and basement membrane maturation in HA-hydrogels. Coordinated multicellular movements on the micron-order were observed to occur in virtually all viable structures. Motion analysis at the cell- and microstructure-level was performed at discrete timepoints to examine both cell-cell and cell-basement membrane dynamics of hS/PC-derived microtissues during microstructure organization. A surprising observation was that the size of salivary microstructures did not increase linearly, rather the motion and expansion of the cells within the structure appeared to exert a force on the basement membrane that first led to its thinning, and then its “popping” to assume a larger diameter (Movie S3). Movie S4 captures expansion events of a pair of salivary microstructures. For example, the basement membrane of a larger microstructures (≥100 μm in diameter) underwent cyclical contractile and tensile phases, evident as “thick” or “thin” basement membranes (Figures 4A,B). A typical “popping cycle” lasted 0.67 ± 0.34 h and led to a volumetric expansion of 42.8 ± 8.3% (*n* = 3) that was assessed by taking measurements of the major and minor axes in individual frames acquired from time-lapse imaging for each microstructure. To determine how microstructures grow at a cellular level, we examined Ki67 expression and found that the vast majority of dividing cells reside at the periphery of the multicellular structures, immediately adjacent to the basement membrane where they are expected to apply compressive force (Figure 4C).

Figure 4D shows how the thickening and thinning phases of the basement membrane changed with time. Contractile or “thick” phases (noted as 1: shaded red) alternated with tensile or “thin” phases (noted as 2,3: shaded blue). Prior to “popping,” a progressive thinning of the basement membrane was seen, eventually leading to its failure and a yielding of its tensile strength as the structure expanded volumetrically. Figure 4E is a schematic that illustrates proposed interactions between the basement membrane and integrin subunits during contractile (phase 1) and tensile events (phases 2–3). Forces exerted on the basement membrane are large enough to break perlecan spot-welds between laminin and collagen IV networks causing subsequent remodeling of the basement membrane.

### Cell-Basement Membrane Interactions: Dependence on α_1_β_1_ Integrin

To investigate the dependence on integrin interactions for dynamic behavior of microstructures within a hydrogel microenvironment, we used a Cy3-tagged siRNA specifically targeted to β_1_ integrin. Not surprisingly, early β_1_ integrin siRNA treatment on single-cells in hydrogel prevented hS/PC microstructures from forming, while scrambled Cy3-tagged siRNA treatment on single-cells in hydrogel yielded microstructure formation through days 7–8 post-treatment (Figure S7). To study the role of β_1_ integrin in microstructure motility, loss of function experiments was undertaken after microstructure formation had already occurred and time-lapse recordings were performed for various treatment groups.

**Figure 5:**
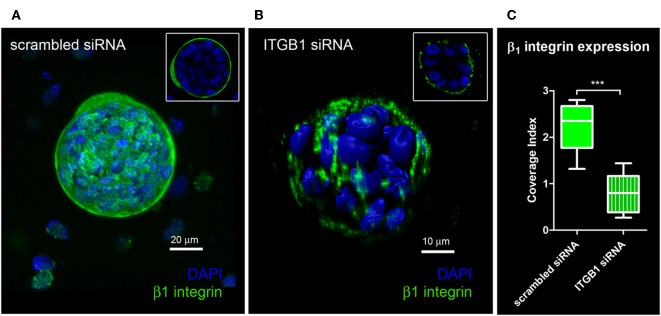
Quantification of knockdown of β_1_ integrin in hS/PC microstructures. hS/PC microstructures formed in HA-based hydrogels and were transfected with scrambled siRNA or ITGB1 siRNA. Immunocytochemistry was used to determine β_1_ integrin expression and spatial distribution of β_1_ integrin 46 h post-transfection. Image analysis software was used to quantify β_1_ integrin expression at microstructure surfaces from confocal micrographs and coverage indices were calculated for scrambled siRNA **(A)** and ITGB1 siRNA **(B)** groups where a 63% reduction in β_1_ integrin expression at microstructure surfaces was quantified **(C)**.

Quantification of knockdown of β_1_ integrin in hS/PC microstructures treated with scrambled siRNA or ITGB1 siRNA was performed in hydrogels using immunocytochemistry to capture spatial distribution of β_1_ integrin expression. In the scrambled siRNA group, β_1_ integrin expression was continuous and surrounded each microstructure's surface (Figure 5A). A discontinuous or “patchy” distribution of β_1_ integrin surrounded each microstructure in the β_1_ integrin knockdown group (Figure 5B). A 63% reduction in β_1_ integrin expression at the surface of hS/PC microstructures was achieved from ITGB1 siRNA treatment in hydrogels (I_C_ = 0.80 ± 0.13, *n* = 10) compared to the group treated with scrambled siRNA (I_C_ = 2.22 ±0.16, *n* = 10) (Figure 5C). Quantification of knockdown using siRNA targeting ITGB1 in hS/PC monolayers was also performed by Western blot (Figure S8). Immunocytochemistry proved to be the more sensitive approach for quantifying β_1_ integrin knockdown and is consistent with the loss of function results obtained from time-lapse imaging experiments.

**Figure 6:**
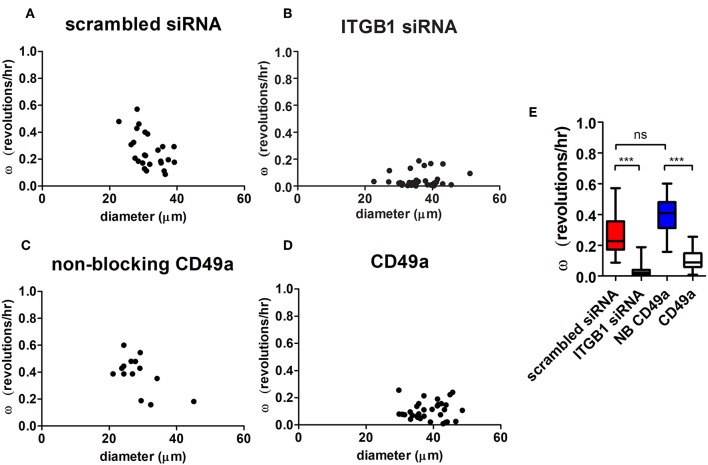
Integrin inhibition diminishes microstructure motility in HA-based hydrogels. Angular velocities (ω) of hS/PC microstructures were plotted with respect to size **(A–D)**. The ω of hS/PC structures treated with siRNA targeting β_1_ integrin demonstrated an 84% mean reduction in ω compared to its control (*p* < 0.0001). Organized hS/PC structures treated with integrin blocking antibody CD49a resulted in a 73% reduction in ω when compared to its non-blocking CD49a control (*p* < 0.0001) **(E)**. The ω of hS/PC structures treated with scrambled siRNA and non-blocking CD49a control groups were not significantly different from each other, *p* = 0.23.

Angular velocities were calculated in both treated knockdown and scrambled control conditions. Organized hS/PC structures treated with β_1_ integrin siRNA demonstrated an 84% mean reduction in ω (ω = 0.04 ± 0.01 rev/h, *n* = 37) compared to the scrambled control (*p* < 0.0001) (Figures 6A,B). Complete arrest of motility was not apparent because a few cells were still motile within the microstructures. Figure S9 shows distribution of Cy3-tagged siRNA in hS/PC microstructures before time-lapse experiments. Acute treatment on hS/PC microstructures with either scrambled siRNA (Figure S9A) or ITGB1 siRNA (Figure S9B) yielded high transfection efficiency where each cell within all microstructures contained Cy3-tagged siRNA. These images were taken prior to each siRNA live-imaging experiment to ensure presence of siRNA in each hS/PC microstructure.

To further define the integrin involved in spinning behavior, hS/PC clusters were treated with a blocking antibody that stabilizes the inactive conformation of α_1_ integrin subunit (CD49a) its partner β_1_ integrin, which would normally complex with laminin and collagen IV. This treatment resulted in a 73% reduction in ω (ω = 0.11 ± 0.01 rev/h, *n* = 32) when compared to the non-blocking CD49a control (*p* < 0.0001), (Figures 6C–E). The reduction of β_1_ integrin activation with blocking α_1_ integrin subunits and direct interactions with the basement membrane significantly reduced the rotational motion observed by the perturbed microstructures. Treatment with Volociximab, a clinically used high-affinity monoclonal antibody that specifically binds to α_5_β_1_ integrin (Ricart et al., 2008), decreased ω (ω = 0.11 ± 0.01 rev/h, *n* = 54) to levels similar to those observed when α_1_ integrin subunit was targeted. The ω of hS/PC structures treated with scrambled siRNA and without treatment were not significantly different from each other, *p* = 0.23, nor were the non-treated and non-blocking CD49a control groups (Figure 6E).

## Discussion

### Microstructure Assembly and Basement Membrane Formation

The pattern and sequence of basement membrane protein production that we observed for hS/PC microstructures in HA-hydrogels was comparable to that seen in embryonic development, wherein laminin is secreted before collagen IV or perlecan (Huang et al., 2003). This finding helps validate the use of the HA-hydrogel platform for study of cell and matrix dynamics as they occur in salivary gland development. Interactions between cells and basement membrane depend on adhesion molecules and surface receptors, primarily integrins (Loducca et al., 2003; Lourenço and Kapas, 2005; Laine et al., 2008), and thus these molecules typically are expressed concordantly with the basement membrane components during embryogenesis (Li et al., 2003). Unlike in the salivary gland, where formation of the gland involves growth and branching morphogenesis originating from a rudimentary salivary bud, in HA hydrogels, microstructures originate from growth from a single hS/PC or merging of adjacent hS/PC microclusters (Pradhan-Bhatt et al., 2013). In HA hydrogels, hS/PCs secrete ligands including laminin, collagen IV, and perlecan that together direct matrix-mediated signaling to establish the apical-basal axis in polarizing secretory epithelium similar to that which forms *in vivo* (Yu et al., 2005; Overeem et al., 2015). This signal transduction is initiated by HA receptors (Spicer and Tien, 2004) and integrins (Hynes, 2002) interacting with their respective ligands to regulate cell cycle, adhesion, differentiation, as well as motility during microstructure growth and organization. These integrin-mediated interactions with basement membrane proteins precede both epithelial morphogenesis (Rebustini et al., 2007) and polarity in developing epithelial tissues (Haigo and Bilder, 2011; Lerner et al., 2013). The observation that hS/PCs secrete their own ECM to promote homeostasis and survival, in a hydrogel naïve of murine extracellular matrix proteins and growth-factors, further supports hS/PCs' role in human tissue engineering applications. Key understanding of hS/PCs ability to maintain key cell machinery for secretory function (Baker et al., 2010) in modifiable hydrogel culture systems (Shubin et al., 2015, 2017) is now of critical importance and continues to challenge us in the field of translational tissue engineering.

A survey of integrins previously reported to be expressed in salivary gland led us to examine the role of β_1_ integrin that is known to be involved in acquisition of polarity in dynamic microstructure formation (Akhtar and Streuli, 2013). A key finding from our work was that the initial deposition of matrix proteins, first laminin, then collagen IV, and finally perlecan, along with expression of the β_1_ integrin, is required for *de novo* basement membrane maturation, and precedes epithelial cell polarization and lumen formation needed for vectorial secretion of salivary components.

In microstructures, perlecan/HSPG2 was deposited with the laminin and collagen IV networks of the organizing *de novo* basement membrane similarly to the way it would in salivary compartments in native tissue (Figure S6). The perlecan distribution that we observed within the basement membrane was not as continuous, seen in Figures 2G–I, as *de novo* laminin or collagen IV networks. Regions of discontinuity could offer spatial opportunities for branching morphogenesis, cleft formation, and interactions with stromal elements to occur. Access to hS/PC derived epithelial microstructures attached to the basement membrane could also encourage integration with connective tissue or support cells, i.e., vascular, neuronal, or immune cells after hydrogel implantation. During salivary microstructure maturation, assembled basement membrane proteins recruit β_1_ integrin-mediated adhesion complexes to the cell surface where they provide survival signals, support motility, and favor additional ECM secretion. Lacking these signals, it is thus not surprising that the majority of cell death that we observed after cells were encapsulated occurred within the single cell population shortly after encapsulation. Once matrix secretion commenced, high viability was observed throughout the rest of the culture period.

### “Spinning” as a Way to Deposit Basement Membrane

Within our tunable HA-based hydrogel system (G' range ~60–300 Pa) (Ghosh et al., 2005; Prestwich, 2011), basement membrane deposition and organization was found to be dynamic and restricted to the microstructure's periphery in 3D. Basement membrane proteins are actively secreted on planar culture systems (E~3 GPa) (Callister, 2000), but do not provide organizational cues and are nine orders of magnitude stiffer when contrasted with most hydrogel systems. However, a laminin- or elastin-modified synthetic 2D scaffold system (E~1–20 MPa), although 10^6^ times stiffer than our hydrogels, triggered tight junction formation and polarization in monolayers of acinar and ductal cells (Cantara et al., 2012; Foraida et al., 2017) reinforcing the role of spatial cues from the basement membrane on reorganizing salivary epithelium.

As salivary hS/PC microstructures matured and were enveloped by an organizing basement membrane, spontaneous global revolutions were observed. Other hydrogel culture systems used by others to encourage epithelial cell growth and organization also revealed coordinated dynamics during cellular reorganization. For example, primary breast cancer cells harvested from murine tumor cells were cultured in rodent Matrigel™. Matrigel™-based culture systems consist of self-assembling basement membrane networks, and these primary murine tumor cells produced multicellular assemblies (Tanner et al., 2012) visibly similar to those produced in our HA-hydrogels. A key difference between our HA-hydrogels and Matrigel™ systems is that our HA-hydrogels are protein-free, thus any proteins present in the basement membrane were produced *de novo* by the encapsulated cells themselves, and were not supplied by the hydrogel system.

3D assembly of mammary and kidney cells in Matrigel™ were reported to exhibit full revolutions of mammary gland acini and MDCK cell cysts (Tanner et al., 2012; Wang et al., 2013). Revolutions were tracked during the fibrillar organization of the surrounding basement membrane. A separate study confined MDCK cell cysts to a micropatterned array, to show they rotated when soluble Matrigel™ components were made available (Rodríguez-Fraticelli et al., 2012). Those MDCK cell “cysts” established basement membranes and formed lumens post-rotation indicating that basement membrane formation and organization preceded polarization. In developing *Drosophila* eggs, global revolutions established a polarized corset of collagen IV matrix to govern elongation (Haigo and Bilder, 2011). Thus, this coordinated multicellular motility and likely mechanical feedback from the microenvironment is likely to be associated with secretion and organization of *de novo* basement membranes surrounding multicellular structures. It should be noted that unlike the process of salivary gland formation during embryonic development, where the bud is “anchored” to the stalk and full range of rotation is limited, the unanchored hS/PC derived microstructures in hydrogels are capable of full 360° rotations.

### Features of Basement Membrane That Support Tissue Expansion and Morphogenesis

We found that HA-hydrogels encouraged the dynamic assembly and movement of microstructures exhibiting coordinated cell-cell and cell-matrix adhesion behavior reminiscent of salivary cell movements during morphogenesis (Lafrenie and Yamada, 1998). This movement is thought to occur via mechano-chemical coupling similar to that previously reported in other systems (Ringer et al., 2017). The basement membranes of larger, mature microstructures were evident at the boundaries between cluster and hydrogel directly interacting with the outermost hS/PCs. This connected hS/PC cell complex could participate in propagating intercellular signals either directly or indirectly using co-signaling mechanisms established throughout the entire microstructure. These hS/PC cell complexes in HA hydrogels are not restricted by the organized basement membrane and will continue to grow into larger structures within growth permissive hydrogel matrices. The hydrogels used to study reorganization dynamics (G' ~ 200 Pa, E~0.6 kPa) had mechanical properties comparable to embryonic tissue (E ~ 0.1 kPa) (Peters et al., 2015), where ν = 0.5 and E = 2G'(1+ν). All microstructures exhibited a tightly organized basement membrane and rarely exceeded diameters >50 μm, similar to dimensions of acinar structures in normal human tissue (E ~ 2 kPa) where acinar diameters range between 30 and 60 μm (not shown). These mechanosensitive interactions thus directly influence the size and shape of these reorganizing salivary microstructures. We have shown salivary hS/PCs require specific integrin binding complexes to achieve the coordinated reorganization within HA-based hydrogel culture systems. With increased knowledge of the effect of stiffness and bioactive potential of our tunable hydrogel on hS/PC growth and reorganization, we may now further design the hydrogel composition to encourage and reveal hierarchical complexities of the growing salivary epithelium.

### Interactions Between Growing Structures and Basement Membranes Favor Non-linear Expansion

**Figure 7:**
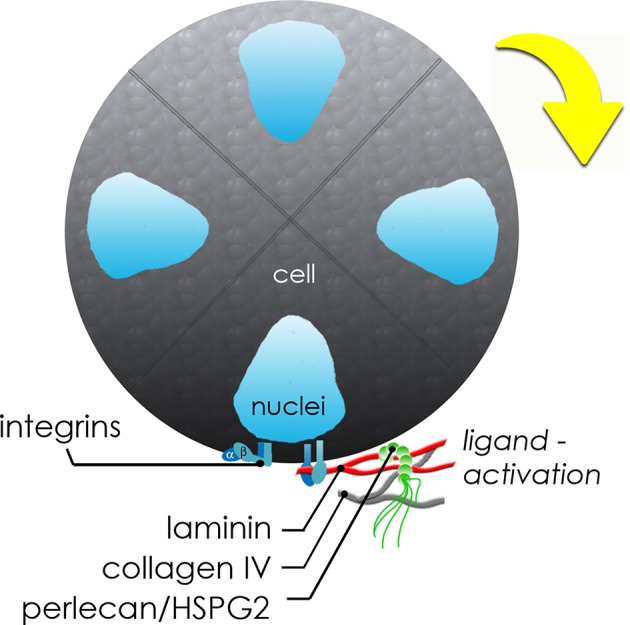
Schematic of molecular interplay during *de novo* microstructure organization. Secreted basement membrane proteins are directionally secreted and organized by hS/PCs at the periphery of the growing microstructure. The initial growth and coordinated movement during this maturation phase depends on β_1_ integrin activation by hS/PC-secreted ligands: laminin, collagen IV, and perlecan/HSPG2. Adhesive interactions between integrins on cells and matrix or hydrogel networks surrounding them can generate traction forces large enough to drive coordinated revolutions of entire multicellular structures.

In Figure 7, we present a proposed model to describe the observed interactions of the basement membrane components that can produce changes in local tensile and adhesive interactions with surface integrins that can occur during microstructure rotation and expansion. Integrin-mediated complexes with basement membrane components generate forces that influence all adjacent cells within the microstructure. If the basement membrane surrounding the microstructure is treated as a thin walled vessel under internal pressure from increasing cell numbers, the increase in compressive force on the structure would generate strain on the basement membrane, enough to cause a “thinning” and potential breakage of the basement membrane fabric at its weakest adhesion points, described in Figure 4 and Movie S3. In the case of this basement membrane network, we propose that the relatively low affinity linkages involving perlecan's heparan sulfate chains may be those weakest links because of its mechanical behavior in tension (Wijeratne et al., 2016) and its role as a crosslinker rather than a true part of the polymeric meshwork of the basement membrane. The rupture of the “spot welds” (Behrens et al., 2012) involving perlecan thus are likely to be those sites first to break during the “pop,” allowing for the observed rapid expansion and initiating a new round of basement membrane formation to surround the larger structure.

### Critical Role of Integrin α_1_β_1_ in Supporting Dynamic Movements of Salivary Microstructures

In this study, β_1_ integrin was found to be essential to both the formation and organization of hS/PC microstructures, evidenced by our finding that knockdown cells lacking normal amounts of β_1_ integrin failed to form multi-cellular microstructures. Additionally, even though single hS/PCs could secrete individual matrix proteins prior to microstructure assembly, activated integrins were required to initiate formation of multicellular structures. We specifically found that β_1_ integrin is a key driver of early microstructure assembly and growth. The role of β_1_ integrins, primarily α_1_β_1_, in dynamic movements of formed structures also was clear for spinning and associated deposition of basement membrane components. Knockdown and antibody-blocked microstructures failed to exhibit the dynamic behaviors of normal microstructures. This is entirely consistent with the appearance of this integrin during salivary development during times of peak glandular growth, morphogenesis and expansion (Lafrenie and Yamada, 1998).

In addition to integrins, salivary hS/PCs possess integrin-independent CD44 and RHAMM receptors for HA (Pradhan-Bhatt et al., 2014) and interact directly with HA to promote cell-cell and cell-matrix adhesion during morphogenesis (Spicer and Tien, 2004). Early studies of the basement membrane in murine embryonic salivary glands showed that hyaluronan accounted for ~50% of newly synthesized glycosaminoglycans (GAGs) in the basal lamina (Cohn et al., 1977). The high presence of hyaluronan during development tracks with the abundance of HA-receptor, CD44, expression in human embryonic parotid glands that persist during adulthood (Franchi et al., 2001). These non-integrin signaling receptors influence cell adhesion, proliferation, invasion, and matrix assembly; however, deletion of these receptors does not result in embryonic lethality (Tammi et al., 2002). Likewise, although these receptors can mediate similar processes to those that integrins regulate (Toole, 2001), their expression in our system alone was not sufficient to encourage growth and maturation of hS/PC microstructures, which required production of basement membrane components and associated integrins.

Although this study primarily focused on laminin, collagen IV and perlecan/HSPG2 as major components of the basement membrane, the presence and role of fibronectin in HA-based hydrogel systems also must be explored in future work. Demonstration that coordinated motility of organizing hS/PC microstructures is partially mediated by α_5_β_1_ integrin, which is known to interact with fibronectin, provides evidence that specific cellular machinery required for branching morphogenesis is preserved in hS/PC microstructures cultured in biocompatible and GMP-ready hydrogels. To drive and establish the hierarchical architecture of the salivary gland, a clearer understanding of basement membrane dynamics in the context of maintaining boundaries during homeostasis and breaking boundaries during growth and morphogenesis is desired.

Fibronectin's role during bulk and local reorganization of the basement membrane triggers branching morphogenesis events in developing salivary glands (Larsen et al., 2006; Harunaga et al., 2014). The basement membrane dynamics at the periphery of larger microstructures would encourage cryptic self-assembly sites within fibronectin to be exposed by tension (Zhong et al., 1998) to encourage local morphological changes. Of particular interest, high-affinity binding of fibronectin by β_1_ integrin (Faull et al., 1993) and β_1_ integrin's role in the mechano-chemical “check-point” of salivary gland cleft formation (Daley et al., 2011), highlights the potential formation of sophisticated glandular geometries within our hydrogel systems, an area of current investigation in our laboratory.

## Ethics Statement

This study was carried out in accordance with the recommendations of the Christiana Care Health System Institutional Review Board (IRB)-approved protocols with written informed consent from all subjects. All subjects gave written informed consent in accordance with the Declaration of Helsinki during their pre-operation consultation. The protocol was approved by the IRBs at CCHS, Rice University and UTHealth as well as by the Committee for the Protection of Human Subjects at UTHealth.

## Author Contributions

DW designed and performed experiments and prepared manuscript. RW provided cell source and clinical advisor. DW, RW, DH, and MF-C provided scientific and manuscript writing contributions.

### Conflict of Interest

The authors declare that the research was conducted in the absence of any commercial or financial relationships that could be construed as a potential conflict of interest.
