# Supplementary material for: Dynamic Assembly of Human Salivary Stem/Progenitor Microstructures Requires Coordinated α1β1 Integrin-Mediated Motility
Source: Front Cell Dev Biol. 2019 Oct 16;7:224. doi: 10.3389/fcell.2019.00224 (PMC6843075; doi:10.3389/fcell.2019.00224)
Supplement: Supplementary file 1 [file Table_1.DOCX]

### Table S1: Integrin Subunits Reported in Normal Human Salivary Gland Tissue and Their Specific Ligands

| **Integrin** | **Ligand** | **Reference** |
| --- | --- | --- |
| α_1_β_1_ | laminin-111, - 211; collagen IV; perlecan-1A , -V, -V_c_ | Brown et al. 1997, Ettner et al. 1998, Laine et al. 2008 |
| α_3_β_1_ | laminin-332, - α5; perlecan | Nishiuchi et al. 2006, Rebustini et al. 2007, Laine et al. 2008 |
| α_5_β_1_ | fibronectin | Patel et al. 2006 |
| α_6_β_1_ | laminin-111, - 332, - α5 | Nishiuchi et al. 2006, Rebustini et al. 2007, Laine et al. 2008 |
| β_1_ | perlecan core protein; large perlecan; perlecan V | Hayashi et al. 1992; Battaglia et al. 1993; Brown et al. 1997; Lourenço and Kapas 2005 |
| β_3_ | perlecan core protein; | Hayashi et al. 1992; Loducca et al. 2003, Lourenço and Kapas 2005 |
| α_6_β_4_ | laminin-332, - α5 | Lourenço and Kapas 2005, Nishiuchi et al. 2006, Rebustini et al. 2007, Laine et al. 2008 |
