# Supplementary figures and images for: Dynamic Assembly of Human Salivary Stem/Progenitor Microstructures Requires Coordinated α1β1 Integrin-Mediated Motility
Source: Front Cell Dev Biol. 2019 Oct 16;7:224. doi: 10.3389/fcell.2019.00224 (PMC6843075; doi:10.3389/fcell.2019.00224)

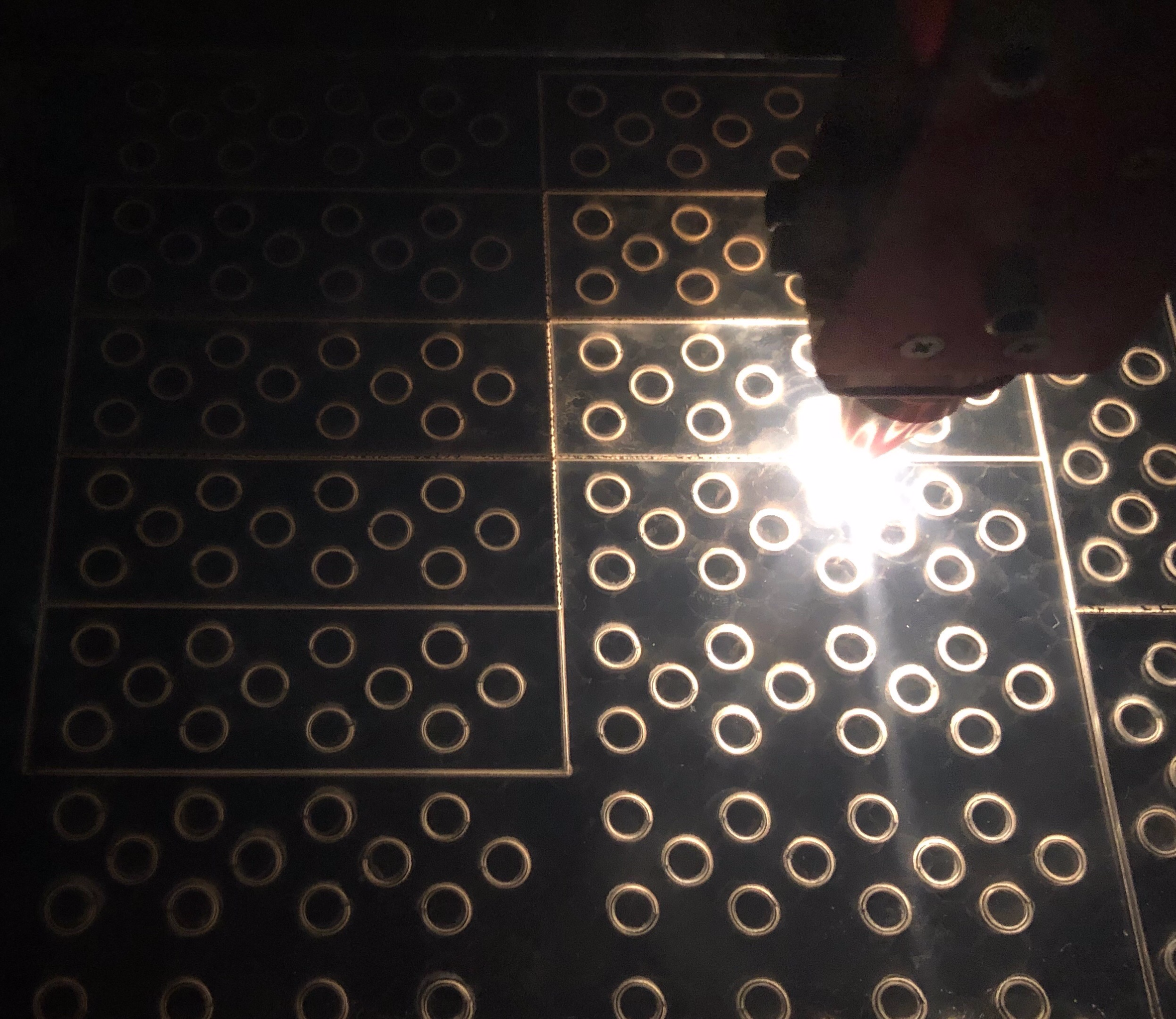

Supplement: Supplementary file 6 [file Image_1.PNG]

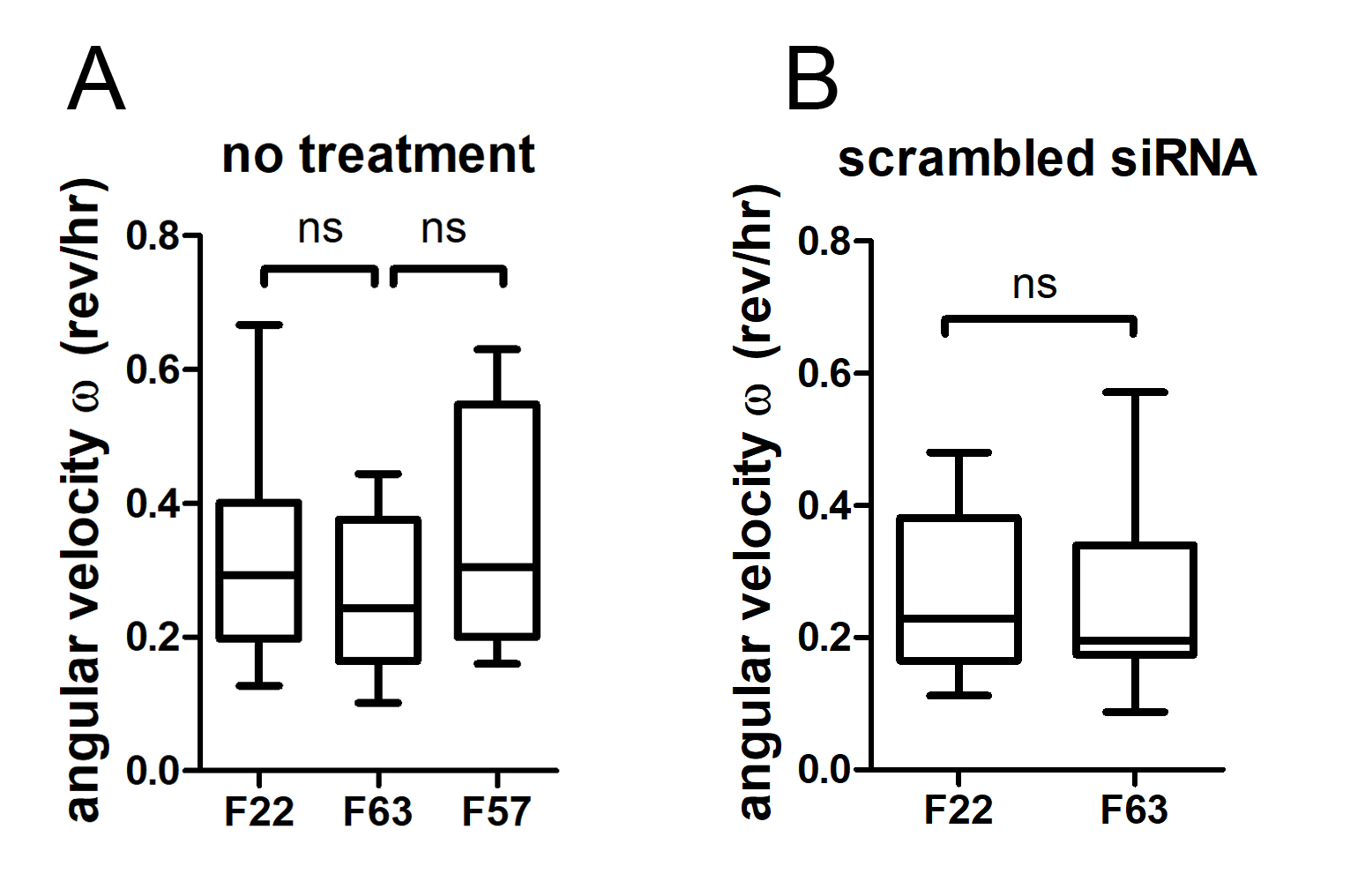

Supplement: Supplementary file 7 [file Image_2.TIF]

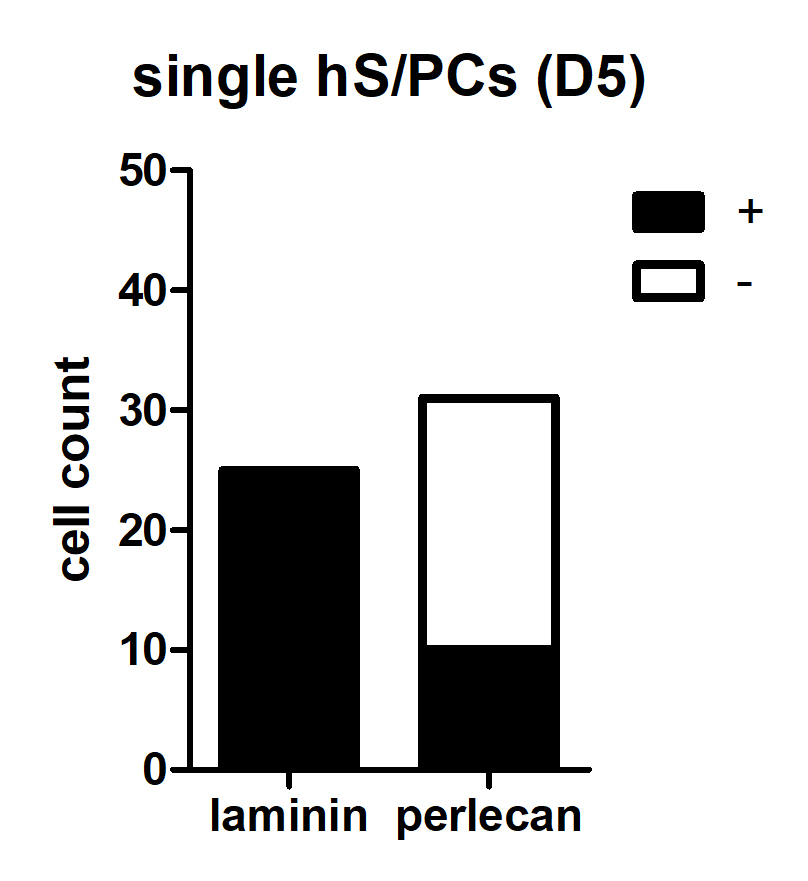

Supplement: Supplementary file 8 [file Image_3.JPEG]

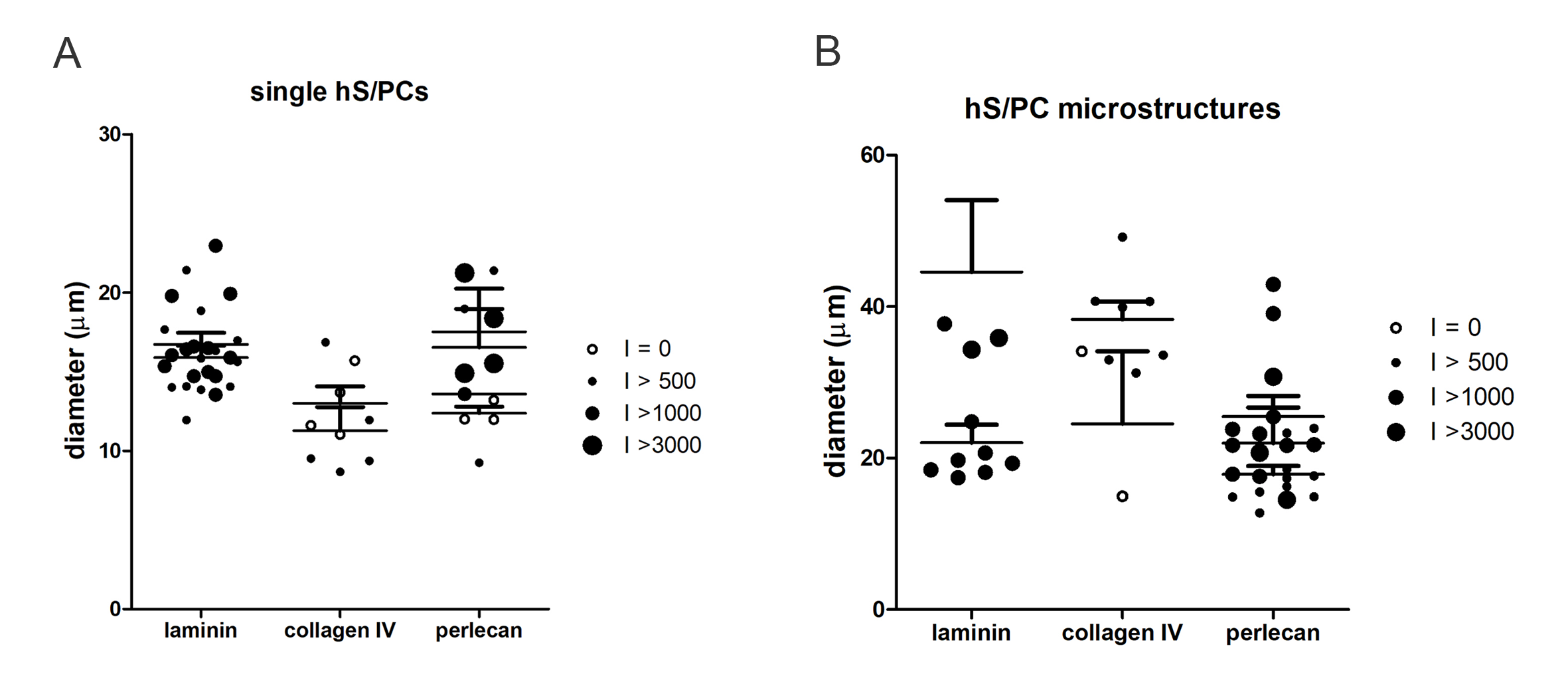

Supplement: Supplementary file 9 [file Image_4.TIF]

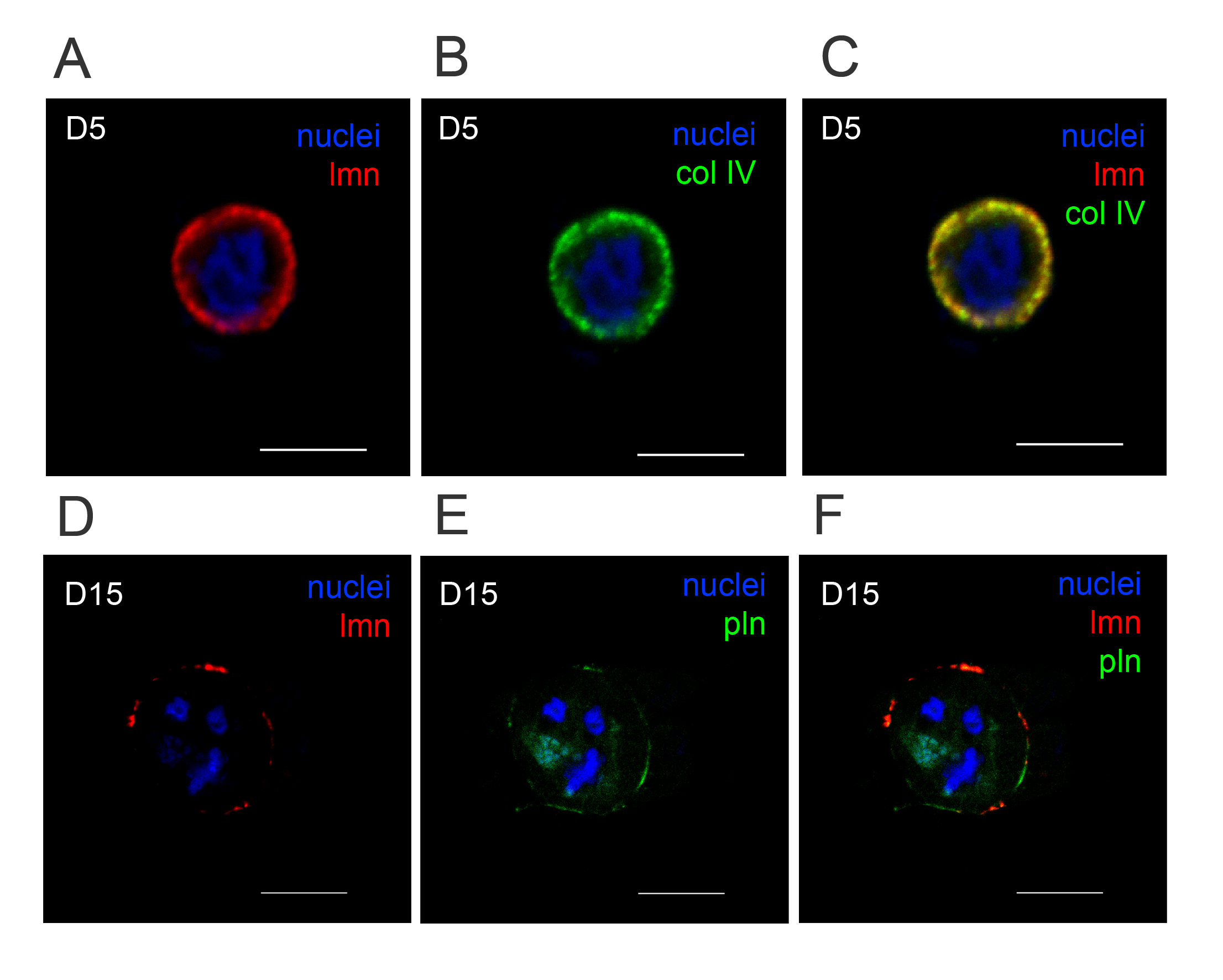

Supplement: Supplementary file 10 [file Image_5.TIF]

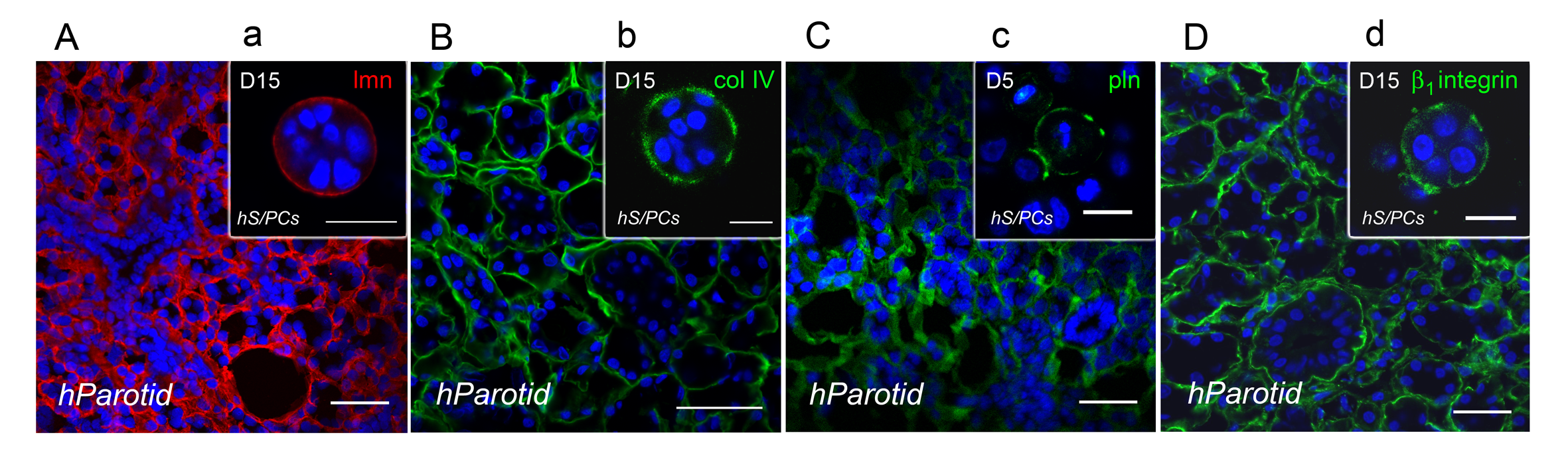

Supplement: Supplementary file 11 [file Image_6.TIF]

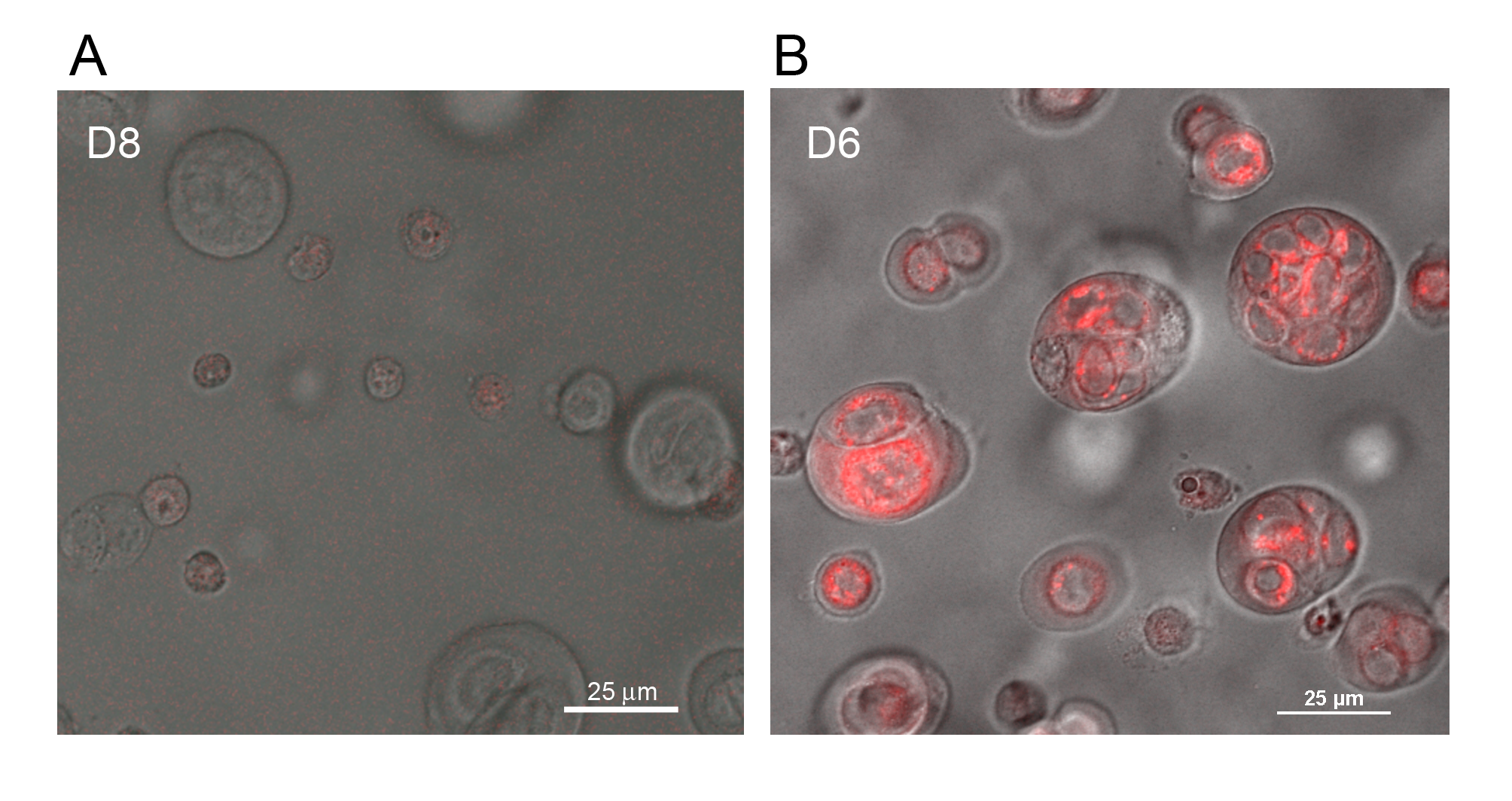

Supplement: Supplementary file 12 [file Image_7.TIF]

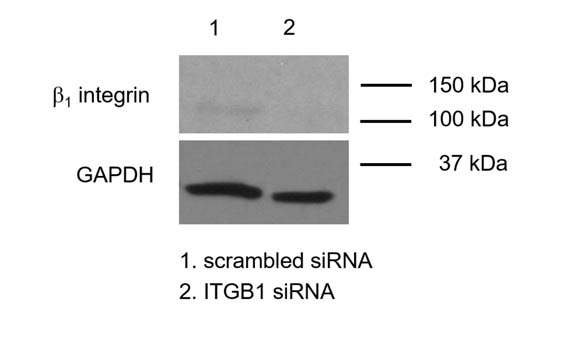

Supplement: Supplementary file 13 [file Image_8.JPEG]

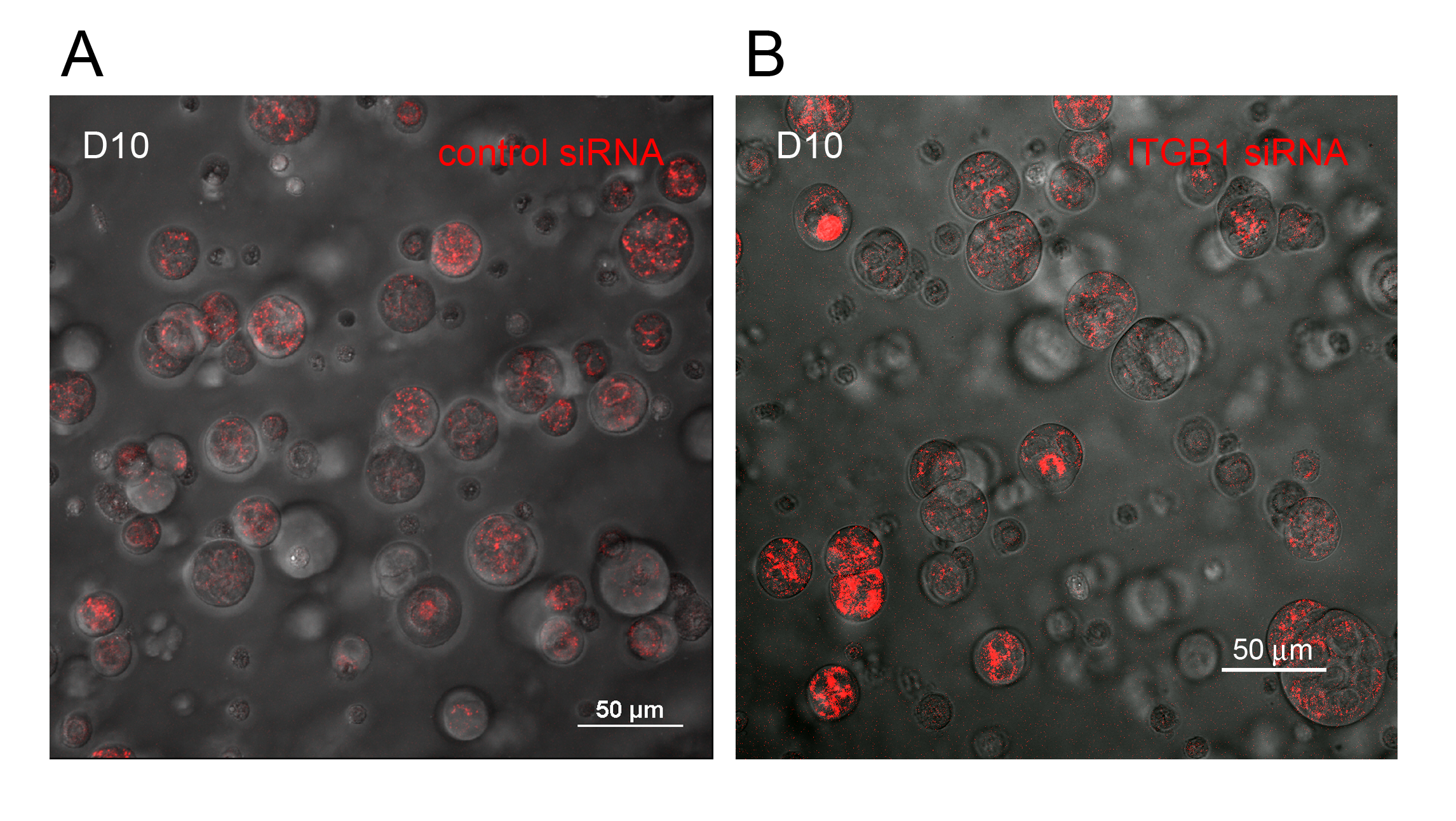

Supplement: Supplementary file 14 [file Image_9.TIF]
