# Supplementary material for: Dynamic Assembly of Human Salivary Stem/Progenitor Microstructures Requires Coordinated α1β1 Integrin-Mediated Motility
Source: Front Cell Dev Biol. 2019 Oct 16;7:224. doi: 10.3389/fcell.2019.00224 (PMC6843075; doi:10.3389/fcell.2019.00224)
Supplement: Supplementary file 15 [file Data_Sheet_1.PDF]

Figure S1. Laser-cut molds made from PDMS sheets.
PDMS molds were designed and used to routinely create hydrogels of the same geometry and volume. These PDMS molds are autoclavable and may be cleaned and reused > 50 times with good fidelity. To make PDMS molds, PDMS sheets were poured and cured upon transparency sheets within a leveled tray to a targeted thickness of 2 mm. A second transparency sheet was placed on the other side of the cured PDMS prior to transportation and laser cutting. The cut-file was designed on Adobe Illustrator to cut arrays of 6 mm circles to fit microscope slides (25 mm x 75 mm). Laser power and speed were optimized for vector cutting each PDMS sheet on a Universal X-660 Laser Cutter.

Figure S2. Structure dynamics of microstructures forming from three different patient specimens were similar.
No significant differences in angular velocities were identified when using cells from three donors (F22, F63, and F57) when data from no treatment conditions was assessed (scrambled siRNA, $p = 0.93$; untreated, $p = 0.55$), so data from control and treated conditions was analyzed together.

Figure S3. Early laminin and perlecan deposition in single hS/PCs.
All single hS/PCs in culture express laminin and 32% express perlecan when evaluated after 5 days in hydrogel culture. This illustrates the early hS/PC-secreted basement membrane proteins at the single cell state beginning with laminin as well as perlecan.

Figure S4. Basement membrane protein expression is secreted at the early single-cell state and is maintained throughout various stages of growth.
Extracellular matrix proteins are quantified surrounding single hS/PCs and hS/PC microstructures and plotted with respect to diameter and fluorescence intensities *I* (A.U.). Laminin, collagen IV, and perlecan/HSPG2 are present at various stages of growth and interact with each other to form a basement membrane.

Figure S5. Early secretion of basement membrane proteins.
Single hS/PCs secrete laminin, collagen IV, and perlecan to establish cell survival signals and initiate basement membrane formation and microstructure organization. Laminin and collagen IV are shown surrounding a single cell at D5 in culture (A-C) and laminin remains at the periphery at the microstructure after 15 days in culture accompanied by perlecan (D-F). Scale bars = 10 μm for A-C and 20 μm for panels D-F.

Figure S6. Microstructures in hydrogels form basement membranes and express β_1 integrin similar to those in native salivary tissue.
Laminin (Panel A), collagen IV (Panel B), and perlecan/HSPG2 (Panel C) separate salivary compartments in human parotid gland tissue. Basement membrane proteins similarly form around the salivary compartments of *de novo* forming salivary hS/PC microstructures (Panel insets a-c). β_1 integrin expression patterns are similar in human parotid tissue (Panel D) and in hS/PC microstructures (Panel d). Panels A-D, scale bar = 50 µm; Panel insets a-d, scale bar = 20 µm.

Figure S7. Early siRNA treatment prevented microstructures from forming.
The investigation of early integrin interactions on microstructure growth within a *de novo* microenvironment was accomplished using a Cy3-tagged siRNA targeted to β_1 integrin. As expected, early β_1 integrin siRNA treatment of single-cells in hydrogel prevented hS/PC microstructures from forming (A) while scrambled Cy3-tagged siRNA treatment of single-cells in hydrogel yielded growing microstructures 7-8 days post-treatment (B), To investigate β_1 integrin-mediated motility in mature microstructures, siRNA treatment was performed after microstructure formation.

Figure S8. Quantification of knockdown of β_1 integrin in hS/PCs
Monolayers of hS/PCs were cultured in a 6-well plate until 60-70% confluent. Cells were either transfected with scrambled siRNA or ITGB1 siRNA for 6 hrs and collected by 72 hrs post-transfection. Primary antibodies anti-integrin β_1 and GAPDH (loading control) were used to determine β_1 integrin expression in both groups to determine knockdown efficiency of ITGB1 siRNA. This β_1 integrin primary antibody was also used to quantify β_1 integrin knockdown using immunocytochemistry in hydrogels, **Figure 5**.

Figure S9. Encapsulated hS/PC microstructures treated with Cy3-tagged siRNA
Acute treatment on hS/PC microstructures with either scrambled siRNA (A) or ITGB1 siRNA (B) yielded high transfection efficiency where each cell within all microstructures contained Cy3-tagged siRNA. These images were taken prior to each siRNA live-imaging experiment to ensure presence of siRNA in each hS/PC microstructure.

Movie S1. Example of manual cell-separation using IMARIS Software. Manually selecting and splicing multicellular structures into single cells was performed for clusters that were erroneously counted as a multicellular cluster.

Movie S2. 'Spinning' salivary hS/PC microstructures. Coordinated adhesive interactions between salivary hS/PCs and the hS/PC-secreted basement membrane generated traction forces large enough to drive observed revolutions within the hydrogel.

Movie S3. Growth Dynamics of a Salivary hS/PC Microstructure. Time-lapse imaging of a microstructure with a diameter of 136 μm captured a volumetric expansion of 33.4% that occurred in 1.3 hrs.

Movie S4. Growth Dynamics of a Pair of Salivary hS/PC Microstructures Time-lapse imaging of two microstructures with diameters of 167 μm and 74 μm captured respective volumetric expansions events of 36% within 25 min and 59% within 15 min.

Table S1. Integrin Subunits Reported in Normal Human Salivary Gland Tissue and Their Specific Ligands. Published literature was consulted to identify the integrin subunits reported to be present in normal human salivary tissue. The summary of these findings is presented in this table and includes key studies identifying binding interactions between these integrins and their ligands: laminin, collagen IV, and perlecan/HSPG2.
